# Enhancing Patients’ Informed Consent Through AI: Systematic Review

**DOI:** 10.2196/93501

**Published:** 2026-09-10

**Authors:** Said Dababneh, Nicole Hébert, Laurence Meloche, Nadine Dababneh, Preslava Aleksieva, Issam Tanoubi

**Affiliations:** 1 Centre de Recherche en Pédagogie de la Santé, Faculty of Medecine, Université de Montréal, Montréal, QC, Canada; 2 Department of Anesthesiology and Pain Medicine, Hôpital Maisonneuve-Rosemont, 5415, boulevard de l'Assomption, Montréal, QC, H1T 2M4, Canada, 1 5819996216; 3 Faculty of Medecine, Université de Montréal, Montréal, QC, Canada

**Keywords:** AI, large language models, ChatGPT, informed consent, patient education, Preferred Reporting Items for Systematic Reviews and Meta-Analyses, PRISMA

## Abstract

**Background:**

Informed consent is a cornerstone of medical ethics, ensuring that patients understand the risks, benefits, and alternatives of procedures before making health care decisions. However, challenges such as complex medical language, time constraints, and variations in patient literacy often hinder comprehension. Recent advancements in AI offer new opportunities to improve the informed consent process.

**Objective:**

This systematic review aims to assess AI’s effectiveness in enhancing patient understanding and decision-making during the informed consent process.

**Methods:**

Following the PRISMA (Preferred Reporting Items for Systematic Reviews and Meta-Analyses) guidelines, a comprehensive literature search was conducted in PubMed, Embase, and the Cochrane Library to identify studies published in the past 5 years on AI’s role in informed consent. Subsequently, the reference lists of selected papers were manually reviewed to include any additional relevant studies. Descriptive and statistical analyses were conducted to evaluate AI’s effectiveness, along with tests of homogeneity to assess the feasibility of a meta-analysis.

**Results:**

A total of 33 studies published between 2020 and 2025 were included, categorized into 3 domains: AI-generated patient education (n=18, 54.5%), AI-generated consent documentation (n=10, 30.3%), and AI-assisted consent acquisition (n=5, 15.2%). Large language models demonstrated high accuracy, though readability consistently fell below the recommended eighth-grade level. The best-performing model, Copilot, achieved a Flesch-Kincaid Grade Level of 10.59 (±1.22). AI-generated documents improved Flesch Reading Ease Scores by 44% to 122% and reduced required comprehension grade levels by 10% to 47%, and GPT-4 produced significantly more comprehensive consent forms than both Bard Gemini Advanced and human-written documents (*P*<.001), with accuracy improving by 47% between GPT-3.5 and GPT-4.0. Among AI-assisted consent acquisition randomized controlled trials, AI-assisted patients demonstrated significantly better comprehension of procedural risks than with physician-led consent (*P*<.001), improved provider-perceived patient understanding in prevasectomy counseling (8.8±1.0 vs 6.7±2.8; *P*=.047), shorter consultation times (7.7±2.3 min vs 10.6±3.4 min; *P*=.05), lower postconsent anxiety in total knee arthroplasty (Hospital Anxiety and Depression Scale–Anxiety subscale: 10.48±3.84 vs 12.75±4.12; *P*=.04), and greater satisfaction with preoperative education (4.22±0.51 vs 3.43±0.84; *P*<.001).

**Conclusions:**

AI has the potential to improve the informed consent process; however, further research is needed to address ethical concerns and ensure its effective, patient-centered integration into clinical practice.

## Introduction

Informed consent is a fundamental principle in medical ethics, ensuring that patients have a clear understanding of the risks, benefits, and alternatives of a procedure before making any health care decisions [1]. Valid consent must be given voluntarily, with patients being fully informed and given the opportunity to ask questions [2]. However, the informed consent process is often hindered by the use of complex medical language and time constraints, making it difficult for patients to fully understand their options and make truly informed decisions [3].

Recent studies indicate that most patients now rely on the internet and social media for health information; yet fewer than 1 in 3 verify the credibility of these sources [4,5]. This highlights that, despite improvements in internet access, informed consent continues to pose a significant challenge. In this context, recent advances in AI, particularly large language models (LLMs), offer promising solutions to the challenges of informed consent [6]. LLMs, designed to process and generate text, are rapidly evolving and are beginning to be incorporated into various medical fields to enhance patient care [7,8]. AI-driven conversational tools can provide personalized, accurate, and easily accessible information about procedures, improving patient understanding. By enabling individualized interactions, these technologies could empower patients with the knowledge needed to make informed decisions and address gaps in patient education and consent [9-11].

As research into AI’s role in addressing the longstanding challenge of informed consent continues to expand, significant variability exists in methodologies, measured outcomes, and the overall effectiveness of these interventions. To our knowledge, no study has systematically synthesized these findings to extrapolate results and assess the current state of this technology for this purpose.

Therefore, this systematic review aims to examine the role of AI technologies, including LLMs, in the informed consent process across all medical specialties. By identifying current applications and assessing their viability, we seek to determine whether AI can effectively address this challenge. Furthermore, if certain applications prove successful, they could be adapted to other medical procedures, thereby enhancing informed consent practices across various specialties.

## Methods

This systematic review adheres to the PRISMA (Preferred Reporting Items for Systematic Reviews and Meta-Analyses) guidelines (Checklist 1) [12]. The study protocol was registered on the International Prospective Register for Systematic Reviews (PROSPERO; 420250652460).

### Search Strategy

On February 3, 2025, a comprehensive literature search was conducted across PubMed, Embase, and the Cochrane Library to identify relevant studies on the role of AI in the informed consent process. The search strategy included terms related to consent, such as “consent,” “informed consent,” “digital consent,” “patient autonomy,” and “shared decision-making,” combined with terms related to AI, including “artificial intelligence,” “machine learning,” “deep learning,” “large language models,” “ChatGPT,” and “generative AI.” Terms were joined using the Boolean operators “AND” and “OR.” To align with the study’s focus on recent advancements, filters were applied to include only full-text, English-language publications since the last 5 years.

### Study Selection and Eligibility

All identified papers were imported into Covidence, where duplicates were removed. Two independent reviewers assessed titles and abstracts according to the predefined eligibility criteria outlined in Textbox 1***.*** Studies that met the inclusion criteria proceeded to full-text review, with any discrepancies resolved through discussion with a third reviewer. A manual review of the reference list of included papers was conducted to identify additional relevant studies. The selected studies examined the use and impact of AI technologies on the informed consent process, assessing key factors such as patient comprehension, decision-making, and satisfaction. Only peer-reviewed, full-text studies published in English within the past 5 years were included.

Textbox 1. **Inclusion and exclusion criteria of the systematic review.**

**Inclusion criteria**

- Studies exploring the use of AI in the informed consent process, including machine learning, large language models, chatbots, and other AI-driven tools
- Studies evaluating patient comprehension of consent information, decision-making quality (including autonomy and confidence), and satisfaction with the process
- Studies published between 2020 and 2025
- Randomized controlled trials, observational studies (cohort, case-control, and cross-sectional), qualitative research, and mixed methods studies

**Exclusion criteria**

- Studies in which AI models are applied for purposes other than informed consent
- Non-English publications
- Studies without full-text availability
- Letters to the editor, notes, and commentary
- All types of reviews (eg, systematic, narrative, scoping, or integration)

### Data Extraction and Analysis

Data extraction was conducted and tabulated in Microsoft Excel, with the extracted information summarized in tables. Descriptive statistics were performed where relevant. To assess homogeneity, Cochrane chi-squared test (Cochran *Q*), Higgins *I*², and the *H*² statistic were calculated. All statistical analyses were completed using the JASP software (JASP Team 2024, version 0.19.0; macOS Monterey Version 12.7.5).

Because outcome definitions, measurement instruments, and rating scales varied across studies, a single outcome, readability measured by the Flesch-Kincaid Grade Level (FKGL) [13], was reported by a sufficient number of studies (n=5) in a form permitting quantitative pooling (study mean, dispersion, and sample size). For this outcome, between-study heterogeneity was quantified using Cochran Q, Higgins *I*², and *H*², calculated under a fixed-effect inverse-variance model in which each study’s effect was its reported mean and its within-study variance was SD²/n; *H*² was defined as Q/(k − 1) and *I*² as max(0, (Q − *df*)/Q). Accuracy, completeness, and validity were each reported in only 2 studies using comparable, extractable data and were measured with heterogeneous instruments; a heterogeneity statistic is uninformative with so few comparable studies, so these outcomes were synthesized narratively.

### Risk of Bias and Quality Assessment

The methodological quality and risk of bias of all included studies were assessed independently by 2 reviewers using validated, design-specific appraisal tools, with discrepancies resolved through discussion with a third reviewer. Given the heterogeneity of study designs, 3 tools were applied according to the study type. The Cochrane Risk of Bias Tool 2 (RoB 2) was used for the 3 randomized controlled trials (RCTs), evaluating 5 domains: randomization process, deviations from intended interventions, missing outcome data, measurement of the outcome, and selection of the reported result [14]. The Newcastle-Ottawa Scale, adapted for cross-sectional studies, was applied to the 28 observational and cross-sectional studies, assessing selection, comparability, and outcome domains on a scale of 0 to 8 stars [15]. The Mixed Methods Appraisal Tool was applied to the 2 experimental studies, evaluating the clarity of the research question, appropriateness of data collection, description of the intervention, outcome measurement, control of confounders, data completeness, and adequacy of statistical analysis [16]. All quality assessments were conducted independently by 2 reviewers, with discrepancies resolved through discussion with a third reviewer.

## Results

### Search Results

**Figure 1.**
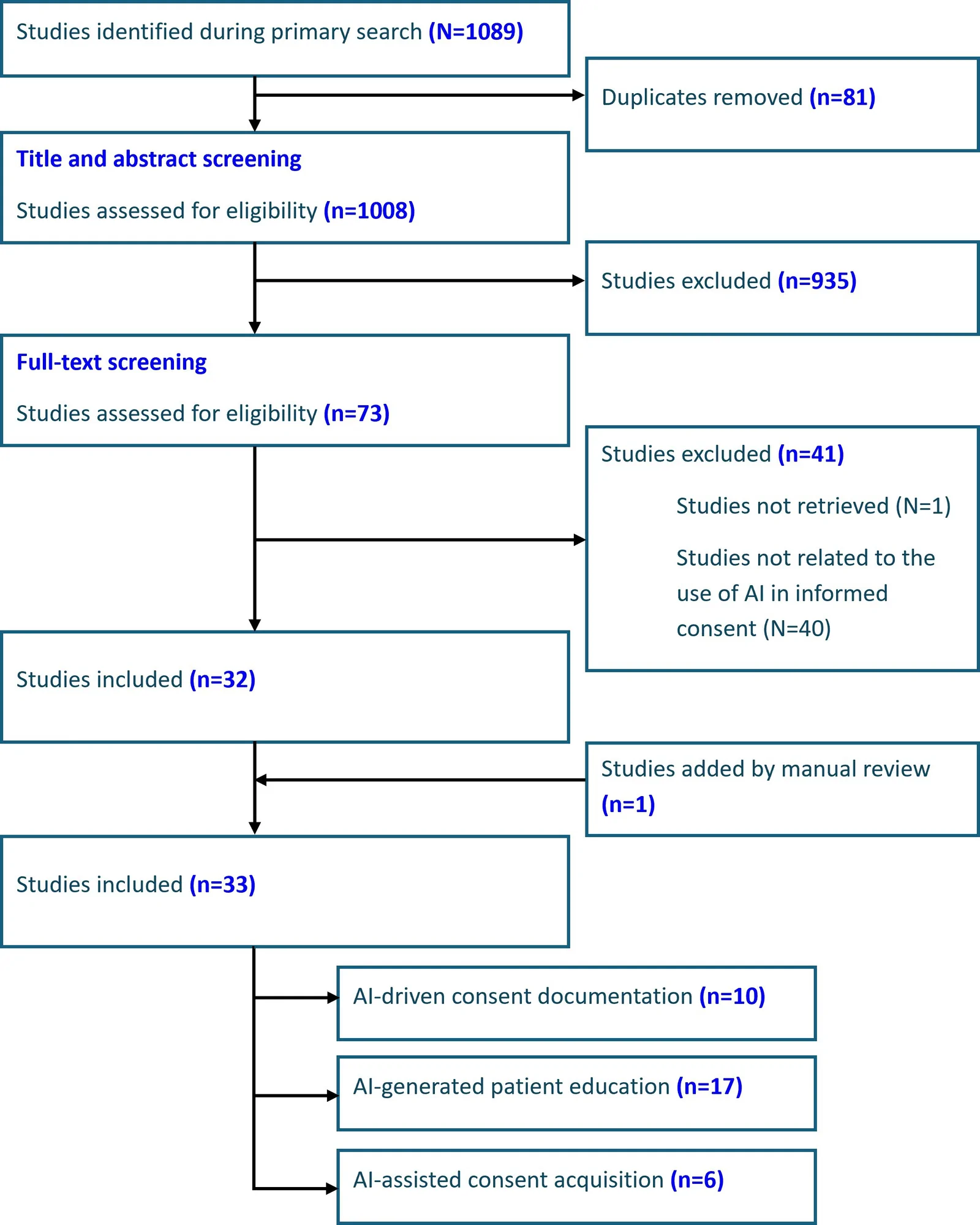
PRISMA (Preferred Reporting Items for Systematic Reviews and Meta-Analyses) flowchart detailing the systematic review process.

A total of 1089 studies were identified and imported into the Covidence platform, where 82 duplicates were removed. After title and abstract screening of the remaining 1007 papers, 70 were eligible for full-text review. Ultimately, 32 studies met the inclusion criteria. An additional relevant study [17] identified through a manual reference list review was included after independent assessment by 2 reviewers confirmed that it met all predefined eligibility criteria, bringing the final total to 33 included studies (Figure 1).

### Study Characteristics

All studies included in this analysis were published within the last 5 years (2020‐2025). The United States accounted for the largest number of studies (n=9, 27.3%), followed by Germany (n=5, 15.2%) and the United Kingdom (n=4, 12.1%). Canada, China, and Turkey each produced 3 studies (n=3, 9.1%), while Australia, Belarus, India, Italy, Japan, and Spain contributed 1 or 2 studies each.

Regarding study designs, cross-sectional studies were the most common (n=21, 63.6%), followed by experimental studies (n=8, 24.2%) and RCTs (n=3, 9.1%). Additionally, 22 of the 33 (66.7%) studies reported no external funding. Detailed characteristics of each study are presented in Table 1.

**Table 1.** Included studies’ characteristics.

Characteristics, study, and year	Country	Funding	Methodology
AI-assisted consent acquisition			
Aydin et al [18] (2023)	Turkey	No	RCT^a^
Chung et al [19] (2024)	Canada	No	RCT
Gan et al [20] (2025)	China	Yes	RCT
Jayakumar et al [21] (2021)	United States	Yes	RCT
Teasdale et al [22] (2024)	United Kingdom	No	Mixed methods study
AI-generated consent documentation			
Brock et al [23] (2024)	United Kingdom	No	Comparative experimental study
Currie et al [24] (2023)	Australia	No	Comparative experimental study
Decker et al [25] (2023)	United States	No	Comparative experimental study
ELSenbawy et al [26] (2026)	Belarus	No	Comparative cross-sectional survey study
Gondode et al [10] (2025)	India	No	Comparative experimental study
Grünebaum et al [27] (2024)	United States	No	Experimental study
Kirchner et al [28] (2023)	United States	No	Comparative experimental study
Patel et al [29] (2024)	United States	No	Comparative experimental study
Shiraishi et al [30] (2024)	Japan	No	Comparative experimental study
Vaira et al [31] (2025)	Italy	Yes	Comparative experimental study
AI-enhanced patient education			
Abou-Abdallah et al [32] (2024)	United Kingdom	No	Cross-sectional survey study
Arora et al [33] (2024)	Canada	No	Cross-sectional survey study
Fahy et al [34] (2024)	Germany	Yes	Cross-sectional survey study
Gabriel et al [35] (2023)	United Kingdom	—^b^	Cross-sectional survey study
Hofmann and Vairavamurthy [36] (2024)	United States	No	Comparative cross-sectional survey study
Kaba et al [37] (2025)	Turkey	No	Cross-sectional survey study
Kerkütlüoğlu et al [38] (2024)	Turkey	No	Cross-sectional survey study
Kienzle et al [39] (2024)	Germany	No	Cross-sectional survey study
Li et al [17] (2024)	China	No	Cross-sectional survey study
Lim et al [40] (2024)	Italy	No	Comparative cross-sectional survey study
Patil et al [41] (2024)	Canada	Yes	Comparative cross-sectional survey study
Schmidt et al [9] (2024)	Germany	No	Cross-sectional survey study
Shah et al [42] (2024)	United States	—	Comparative cross-sectional survey study
Shao et al [43] (2023)	China	No	Cross-sectional survey study
Smith et al [44] (2024)	United States	No	Cross-sectional survey study
Stroop et al [45] (2024)	Germany	No	Comparative cross-sectional survey study
Szczesniewski et al [46] (2024)	Spain	None	Comparative cross-sectional survey study
Trapp et al [47] (2025)	Germany	No	Comparative cross-sectional survey study

a RCT: randomized controlled trial.

b Not available.

### Heterogeneity Assessment

Variability in study methodologies, medical specialties, and outcome measurement scales resulted in substantial heterogeneity across the included studies, preventing a meaningful meta-analysis. Readability, assessed using the FKGL, was the only outcome measured with a sufficiently consistent and comparable metric across a sufficient number of studies (n=5) to allow for the quantitative assessment of heterogeneity [9,10,26,37,38]. For these studies, between-study heterogeneity was extremely high (*Q*=203.06, *df*=4, *P*<.001; *I*²=98.0%; *H*²=50.76). According to conventional Cochrane thresholds, where *I*² values greater than 75% indicate considerable heterogeneity, these findings suggest that nearly all observed variations were attributable to true differences between studies rather than sampling errors.

Other outcomes, including accuracy, completeness, and validity, were each reported in only 2 studies and were assessed using different instruments and scoring systems, preventing meaningful quantitative synthesis. Given the substantial statistical heterogeneity observed, together with the marked clinical and methodological diversity among the included studies, a quantitative meta-analysis was not considered appropriate.

### Quality Assessment and Risk of Bias

Overall, the 3 RCTs were rated as having some concerns across all RoB 2 domains, primarily due to the inherent impossibility of blinding participants and personnel in AI-assisted intervention trials, which represent a structural limitation of this study design rather than an avoidable methodological flaw. Among the 28 cross-sectional studies, 2 were rated as good, 23 as fair, and 3 as poor, with the most common limitations being the absence of a comparator group, nonvalidated outcome instruments, and a lack of evaluator blinding. The 2 experimental studies were rated as moderate, with the most frequent limitations being a lack of a formal control condition and the use of simulated settings. These findings are consistent with the overall moderate methodological quality of the current evidence base for AI applications in informed consent and are reflected in the cautious interpretation of findings throughout this review.

### AI Applications in the Informed Consent Process

The included papers were categorized based on the role of AI in the informed consent process. The first category, *AI-enhanced patient education*, examines AI’s effectiveness in enhancing patient understanding by providing tailored explanations, answering questions, and improving the overall comprehension of medical procedures, including associated risks, benefits, and alternatives. The second category, *AI-generated consent documentation*, focuses on AI’s ability to draft accurate, comprehensive, and simplified consent documents while ensuring clarity and adherence to required standards of validity. Finally, the third category, *AI-assisted consent acquisition*, includes studies that explore AI’s role in directly facilitating and improving the informed consent process by engaging with patients and ensuring comprehension.

### AI-Generated Patient Education

A total of 18 (54.5%) studies examined LLMs’ effectiveness to provide patient education by responding to common inquiries. ChatGPT (all versions combined) was the most frequently evaluated model, appearing in 16 of 18 (88.9%) studies. Other AI models, primarily used as comparators, included Copilot (n=3), Bard (n=2), Gemini (n=2), and Claude (n=2). Surgical specialties accounted for the majority of studies (n=14, 77.8%). Ten studies employed a comparative approach, with 6 evaluating multiple AI models and 4 assessing LLM performance against established sources, such as organization-approved patient pamphlets. Orthopedic surgery and urology were the most frequently studied fields (n=4, 23.5% each), followed by plastic surgery and radiology (n=2 each). The remaining specialties included otolaryngology, ophthalmology, thoracic surgery, spine surgery, oncology, and cardiology, each represented by a single study.

In terms of outcomes, accuracy was assessed in all studies (n=18), while readability was evaluated in 9 studies. Other commonly examined metrics included completeness, reference quality, and relevance (each assessed in 3 studies). The number of questions analyzed varied, with most studies evaluating 20 to 30 questions. The smallest dataset was assessed by Abou-Abdallah et al [32] and Trapp et al [47] (n=6), while Stroop et al [45] examined the highest number of questions (n=139).

Overall, LLMs demonstrated a high level of accuracy in patient education across multiple studies [17,35,37]. This trend was further supported by Shah et al [42], who found that ChatGPT outperformed the Urology Care Foundation’s educational materials, while Stroop et al [45] reported that it enhanced standard informed consent forms by providing 22% more information.

In a study proposed by Hofmann and Vairavamurthy [36], where GPT-4 generated informed consent information for interventional radiology procedures, 84% of physicians rated its accuracy as sufficient, while 85% approved its readability [36]. Interestingly, physician comfort with AI-generated content declined with experience, as an inverse correlation between years in practice and comfort ratings suggested that more experienced physicians were less inclined to rely on AI for this purpose.

However, poor readability was a persistent challenge across LLM-generated responses, with models frequently struggling to produce content at or below the recommended eighth-grade reading level, regardless of the readability test used. In a comparative analysis of multiple LLMs, Lim et al [40] found that Copilot generated the most accessible responses, achieving an FKGL of 10.59 (±1.22), thereby outperforming ChatGPT, Gemini, and Claude. The FKGL is a readability metric that estimates the US school grade required to comprehend a given text, based on factors such as sentence length and syllable count [14]. Meanwhile, Shah et al [42] demonstrated that ChatGPT’s readability could be improved, with its reading grade level decreasing from 12.16 to 7.50 when asked to simplify responses, while maintaining high content quality.

The reliability of information sources is another key concern when using LLMs. Kienzle et al [39] found that while ChatGPT provided excellent or near-excellent information with high interrater reliability (intraclass correlation coefficient=0.79), 37% of its references were fabricated. Similarly, Li et al [17] reported that ChatGPT occasionally included incorrect or outdated citations, highlighting the need for caution and verification. However, Fahy et al [34] observed improvements in this area, noting that GPT-4.0 outperformed GPT-3.5 by citing sources more frequently and offering better external references for patient support and information, suggesting ongoing advancements in LLMs’ ability to provide reliable sourcing.

Table 2 provides detailed information on the AI models used, the medical specialties covered, the number of questions evaluated, the assessment criteria, and the key findings of the included studies.

**Table 2.** Comparison of AI models used for patient education.

Study	AI model	Medical specialty	Comparison source	Number of questions	Assessment criteria	Key findings
Abou-Abdallah et al [32]	GPT-3.5	ENT^a^	ENT UK’s published information	6	Accuracy (DISCERN) Readability (FRES^b^, FKGL^c^, GFI^d^, SMOG^e^)	ChatGPT had poor readability, with FRES scores of 38.9 and 55.1 before and after simplification. Simplified text was 43.6% more readable but 11.6% lower in quality. ENT UK patient information outperformed in both aspects.
Arora et al [33]	GPT-3.5	Orthopedic surgery	Bing Chat AskOE	25	Accuracy Clinical completeness Relevance References Ranking system: 0 (poor) to 100 (best)	AskOE outperformed ChatGPT and Bing (*P*<.001), in all 4 categories (clinical accuracy, completeness, usefulness, and references) and was preferred by reviewers to a significantly greater extent.
Fahy et al [34]	GPT-4.0	Orthopedic surgery	GPT-3.5	23	Accuracy (DISCERN) Readability (FRES, FKGL, GFI, SMOG, Fry Score, Raygor Estimate)	GPT-4.0 had a significantly higher DISCERN score than version 3.5 (48.74 vs 44.59; *P*<.001). Their mean reading grade level showed no significant difference, with neither producing answers at or below the recommended eighth-grade level, regardless of the readability test used.
Gabriel et al [35]	GPT-3.5	Urology	BAUS^f^ patient information leaflet	14	Accuracy Relevance (Global assessment)	78.6% of ChatGPT’s answers aligned with the information in the BAUS patient leaflet, while 92.9% of ChatGPT’s responses were accurate, appropriate, and relevant to patient inquiries.
Hofmann and Vairavamurthy[36]	GPT-4.0	Radiology	None	5	Accuracy Comprehensiveness Readability Physician comfort Conversational tone (5-point Likert scale)	GPT-4’s responses were rated highly for accuracy (mean 4.29, 84% physician approval), readability (4.15, 85%), and conversational tone (4.24, 85%), but were less comprehensive (3.85, 71%) and had lower physician comfort (3.82, 67%). Notably, more experienced physicians were less comfortable with AI-generated consent materials, as indicated by a significant inverse correlation between years in practice and output ratings (*P*=.01)
Kaba et al [37]	GPT-4.0	Radiology	None	25	Accuracy and reliability (5-point Likert scale) Readability (FKGL, FRES, and SMOG)	GPT-4 provided accurate information, with high agreement between 2 radiologists (ICC^g^=0.928) and no significant difference in their assessments (104 vs 109/125, *P*=.244). However, readability was low, requiring advanced education and health literacy (FKGL: 12.51±1.14, FRES: 30.27±8.38, SMOG: 14.46±0.76).
Kerkütlüoğlu et al [38]	ChatGPT (version NS)^m^	Cardiology	None	8	Accuracy and reliability (scale: 1‐10) Readability (FKGL and SMOG)	ChatGPT-generated responses were rated as trustworthy (8.4/10) and valuable (7.9/10) by medical experts, with minimal perceived risk (mean: 2.1/10). However, readability scores were high (FKGL: 13.52, SMOG: 12.49), indicating that a high level of education is required for comprehension.
Kienzle et al [39]	GPT-4.0	Orthopedic surgery	None	50	Accuracy (DISCERN)	ChatGPT’s responses scored above 3 in most categories, often reaching 4. Interrater reliability was high (ICC=0.79), but 37% of the 27 references were fabricated, while only 15% had correct DOI or PMID.
Li et al [17]	GPT-4.0	Plastic surgery	None	8	Accuracy, informativeness, and accessibility (qualitative evaluation)	ChatGPT provided clear and informative responses, aligning with established medical guidelines but offering only generalized advice. While its answers were generally comprehensive, it occasionally included incorrect or outdated references.
Lim et al [40]	GPT-3.5	Plastic surgery	Google Gemini Microsoft Copilot Claude	15	Accuracy (DISCERN and 5-point Likert scale) Readability (FRES, FKGL, CLI^h^)	GPT-3.5 required the highest reading level (FKGL: 13.49±1.35, FRES: 35.22±7.44), making it the least accessible, while Copilot had the lowest FKGL (10.59±1.22) and highest patient-friendliness. Claude had the highest reliability (DISCERN: 54.60±2.23), followed by GPT-3.5 (53.00±2.04), while Copilot excelled in clarity and engagement, scoring the highest on the Likert scale (20/25). Gemini performed the worst in comprehensiveness (Likert: 16/25) and reliability (DISCERN: 49.13±1.77), while Copilot uniquely provided visual aids and hyperlinks, though some were irrelevant or misleading.
Patil et al [41]	GPT-4.0	Ophthalmology	Google BARD	30	Accuracy (5-point Likert scale) Readability (response length)	ChatGPT had significantly higher accuracy ratings than Bard (4.5±0.6 vs 3.8±0.8, *P*<.001). There was no significant difference between ChatGPT and Bard for response length (2104.7±271.4 vs 2441.0±633.9 characters; *P*=.12), while both chatbots lacked information on adverse event.
Schmidt et al [9]	GPT-4.0	Urology	None	20	Accuracy (DISCERN) Readability (FRES and FKGL)	GPT-4 provided moderate-quality information, with DISCERN scores ranging from 2.57 to 2.79 across categories. However, its readability was poor, with FRES between 9.8 and 28.39 and FKGL from 14.04 to 17.41, making it difficult for general patient comprehension.
Shah et al [42]	GPT-4.0	Urology	Educational material from EPIC^i^ and UCF^j^	79	Accuracy (DISCERN, PEMAT^k^, 5-point Likert scale) Readability (FRES, FKGL, GFI, SMOG, CLI, ARI^l^)	ChatGPT provided the highest quality scores (64.33) compared to UCF (61.67) and EPIC (49.5) but had the worst readability, with an average grade level of 12.16 vs 8.44 (UCF) and 5.81 (EPIC). When adjusted for readability (ChatGPT-a), the grade level improved to 7.50 while retaining high quality.
Shao et al [43]	ChatGPT (version NS)	Thoracic surgery	None	37	Accuracy and comprehensiveness (binary qualification based on appropriateness [≥80%] and comprehensiveness [≥50%])	ChatGPT provided appropriate and comprehensive patient education for 92% of responses in both English and Chinese. However, 8% of responses were inadequate, particularly in diagnosing disease symptoms and surgical complications.
Smith et al [44]	ChatGPT (version NS)	Orthopedic surgery	None	60	Accuracy (3-point Likert scale ranging from 1 to 3)	ChatGPT’s responses showed partial to full agreement with expert opinions, with a mean Likert score of 2.43 out of 3. No significant differences were found across 6 subspecialties (*P*=.18).
Stroop et al [45]	ChatGPT (version NS)	Spinal surgery	Standard informed consent form	139	Understandability Accuracy Completeness Specificity Empathy Usefulness Impact on doctor-patient communication Comparison with informed consent forms (Preestablished answer options for each question)	ChatGPT provided largely comprehensible and medically accurate responses, with 97% of spinal surgeons rating the answers as very understandable and 86% considering them satisfactory. It covered 48% of informed consent content while adding 22% of new details. However, 31% of answers were too general, and 1.3% contained serious medical errors.
Szczesniewski et al [46]	ChatGPT (version NS)	Urology	Google BARD Microsoft Copilot	15	Accuracy, thoroughness, clarity, and appropriateness (DISCERN, 5-point Likert scale)	Copilot had the highest DISCERN scores (up to 5/5) but lacked appropriateness, while BARD provided the most accurate responses (≥3/5 for all conditions). For surgical procedures, BARD scored up to 13/15, ChatGPT 8-13/15, and Copilot 4-11/15, with Copilot offering the most citations but the least detailed explanations. Response quality varied between English and Spanish for the same questions.
Trapp et al [47]	GPT-4.0	Radiation oncology	Google Gemini Microsoft Copilot Claude GPT-4.0	6	Accuracy, completeness, and relevance (5-point Likert scale by experts) Readability (FRES) Comprehensibility, accuracy, relevance, trustworthiness, and overall informativeness (5-point Likert scale by patients)	GPT-4, GPT-4o, and Claude AI provided the most complete responses, while Copilot and Gemini were rated as less comprehensive (scores: 4.0‐4.2 vs 2.8‐3.2). Readability was low across all models, with FRES ranging from 24 (GPT-4.0) to 39 (Gemini), yet 94% of patients found GPT-4’s responses easy to understand, 89% found them relevant, 76% trusted the information, and 77% would use it for future medical questions.

a ENT: ear, nose, throat.

b FRES: Flesch Reading Ease Score.

c FKGL: Flesch‐Kincaid Grade Level.

d GFI: Gunning-Fog Index.

e SMOG: Simple Measure of Gobbledygook.

f BAUS: British Association of Urological Surgeons.

g ICC: intraclass correlation coefficient.

h CLI: Coleman-Liau index.

i EPIC: European Patient Information Centre.

j UCF: Urology Care Foundation.

k PEMAT: Patient Education Materials Assessment Tool.

l ARI: Automated Readability Index.

m NS: not specified.

Specialty-specific recommendations could not be established because most specialties were represented by only 1 or 2 studies, which often evaluated different models. The most consistent finding across the literature was the improvement in performance from GPT-3.5 to GPT-4, particularly with respect to accuracy and completeness. However, the contribution of specific technical features to these performance differences could not be determined, as model architectures and version details were inconsistently reported.

A summary comparison of the performance and characteristics of the evaluated LLMs is provided in Table 3*.* However, comparisons across different studies should be interpreted with caution, as results were influenced by variations in medical specialty, prompt design, evaluation methods, and model versions. Therefore, such comparisons are considered hypothesis-generating rather than definitive.

**Table 3.** Large language model (LLM) comparison.

Model	Accuracy/clinical quality	Readability	Completeness	References
GPT-3.5	Moderate	Poor	Moderate	[26,38]
GPT-4/4.0	Highest	Poor	High	[31,44]
Gemini/Bard	Moderate	Poor-moderate	Low	[26,31,44]
Copilot	Moderate-high	Best	Not reported	[33,38,44]
Claude	Moderate	Moderate	Moderate	[33,38,44]

### AI-Driven Consent Documentation

Ten out of 33 (30.3%) papers explored LLM’s ability to produce consent documents. Shiraishi et al [30] evaluated ChatGPT’s ability to generate consent documents for ophthalmic plastic surgery, standing out by incorporating both experts and nonmedical staff in the assessment. Their findings showed no significant difference between evaluations from board-certified plastic surgeons and nonmedical staff.

Currie et al [24] explored the evolving capabilities of ChatGPT over time by comparing version 3.5 with the newer 4.0 version. Their study demonstrated that GPT-4 significantly outperformed its predecessor across all 4 evaluated categories—accuracy, appropriateness, currency, and fitness for purpose—when assessed using a 5-point Likert scale. However, despite these improvements, neither model received an “excellent” (5) rating for any response.

ElSenbawy et al [26] compared GPT-3.5 and Google Gemini in generating pamphlets on deep vein thrombosis, decubitus ulcers, and hemorrhoids. They found no significant difference in accuracy or readability, both requiring a Flesch-Kincaid grade 10 level and scoring 2.33 for reliability on a modified DISCERN scale. However, neither model was compared with an approved reference document. Adding another dimension, Vaira et al [31] conducted the only study comparing multiple LLMs, including Bard Gemini Advanced and ChatGPT, to human-written consent documents. Their findings revealed that GPT-4 consistently produced more accurate and significantly more readable consent forms than those written by humans. Additionally, GPT-4-generated documents were significantly more comprehensive than those created by both Bard Gemini Advanced and human experts (*P*<.001).

Methodological differences, variations in medical specialties, and the subjective nature of expert evaluations resulted in substantial heterogeneity across studies. This high level of variability limited the feasibility of conducting a meta-analysis, even when multiple studies employed the same evaluation metric. Table 4 provides more detailed information, including the original source of the documents, the AI models used, the medical specialty, and the evaluated outcomes.

**Table 4.** Overview of studies on AI models for generating informed consent documents.

Study	Original document source	Generated documents		Medical specialty	Outcomes
		AI model used	Count		
Brock et al [23]	Every Informed Decision Online (EIDO) patient information leaflet for carpal tunnel release	GPT-3.5	2	Hand surgery	Accuracy Readability
Currie et al [24]	GPT-3.5	GPT-4.0	7	Nuclear medicine	Accuracy Appropriateness, Currency Fitness for purpose
Decker et al [25]	Surgeon generated	GPT-3.5	6	General surgery	Accuracy Completeness Readability
ELSenbawy et al [26]	None	GPT-3.5 Google Gemini	6	Multiple	Accuracy Readability
Gondode et al [10]	BPS^a^	GPT-3.5	4	Anesthesia	Accuracy Actionability and understandability Completeness Emotional tone Readability
Grünebaum et al [27]	GPT-4.0 Claude	GPT-4.0 Claude	2	Gynecology	Readability
Kirchner et al [28]	AANS^b^ Rothman Orthopedic Institute Emory Health Massachusetts General Hospital AAHKS^c^ University of California at San Francisco	NS^d^	20	Orthopedic surgery	Readability
Patel et al [29]	ASPS^e^	GPT-4	5	Plastic surgery	Accuracy Completeness Readability
Shiraishi et al [30]	University of Tokyo Hospital	ChatGPT (version NS)	2	Ophthalmic plastic surgery	Accuracy Informativeness Accessibility
Vaira et al [31]	First-year oral surgery resident	GPT-4 Google Bard (now Gemini)	10	Maxillofacial surgery	Accuracy Completeness Readability Validity

a BPS: British Pain Society.

b AANS: American Association of Neurological Surgeons.

c AAHKS: American Association of Hip and Knee Surgeons.

d NS: not specified.

e ASPS: American Association of Plastic Surgeons.

### Readability

Readability was the most frequently assessed outcome, examined in 7 out of 9 studies. Various readability metrics were used, including the FKGL (n=6), Flesch Reading Ease Score (FRES; n=4), Gunning Fog Index (n=3), Simple Measure of Gobbledygook index (n=1), Coleman-Liau Index (n=1), and a 5-point Likert scale (n=1). The use of an LLM significantly improved readability across studies, with FRES increasing by 44% in Kirchner et al [28] and up to 122% in Grünebaum et al [27]. Additionally, the required grade level for comprehension decreased by 10% to 47% in all studies except for Gondode et al [10]. In contrast, Gondode et al [10] found that AI-generated informational documents for 4 common pain medications were less readable than traditional pamphlets, with a 1.9-point increase in FKGL and a 14.3-point decrease in FRES. Nevertheless, their study also included a comparative sentiment analysis, revealing that LLM-generated content was associated with more positive sentiments, whereas traditional documents conveyed a more serious tone and contained more negative sentiments, potentially influencing reader engagement.

### Accuracy, Completeness, and Validity

Accuracy, completeness, and validity were assessed in 7 out of 9 studies to determine whether improved readability compromised content quality, completeness, or document validity. These factors were evaluated using multiple measures, including the Decker et al scale [25], a 5-point Likert scale, and the DISCERN scoring system. The Decker et al scale [25], similar to a 4-point Likert scale, ranges from 0 to 3, categorizing information as incorrect (0), absent (1), incomplete (2), or complete (3). In 5 studies, no significant differences were observed between AI-generated and original documents regarding these metrics. However, Brock et al [23] reported a statistically significant improvement in LLM-generated documents, with a 15% increase (Student *t*=0.014). Similarly, Currie et al [24] found a 47% increase in accuracy between GPT-3.5 and GPT-4.0, reflected by a 0.9-point gain on a 5-point Likert scale. A comprehensive analysis of outcomes is presented in Table 5*.*

**Table 5.** Outcomes of AI-generated patient education documents.

Outcome, evaluation metric, and study	Score, mean (SD)		Score difference (%)
	Original	AI-generated	
Readability			
FKGL^a^			
Brock et al [23]	12.3	7.5	−39
Decker et al [25]	15.3 (2.6)	11.7 (2.4)	−24
Gondode et al [10]	8.3 (0.5)	10.2 (0.5)	23
Kirchner et al [28]	11.3 (1.2)	6 (0.7)	−47
Patel et al [29]	12.5	11.2	−10
FRES^b^			
Gondode et al [10]	62.3 (1.6)	48.0 (3.7)	−23
Grünebaum et al [27]	30.3	67.4	122
Kirchner et al [28]	53.5 (8.9)	76.9 (3.2)	44
GFI^c^			
Decker et al [25]	19.6 (3.1)	15.6 (2.7)	−20
Gondode et al [10]	11.85 (0.9)	13.65 (0.7)	15
Vaira et al [31]	20	ChatGPT: 17.2	−14
Vaira et al [31]	20	Bard: 23.1	16
SMOG^d^			
Decker et al [25]	13.1 (1.4)	11.3 (2.0)	−14
CLI^e^			
Decker et al [25]	14.9 (1.3)	13.0 (1.5)	−13
5-point Likert scale			
Vaira et al [31]	4	ChatGPT: 4; Bard: 4	No statistical difference
Accuracy, completeness, and validity			
DISCERN			
Brock et al [23]	71/80	62/80	15
Decker et al 0‐3 scale			
Decker et al [25]	2.2 (0.4)	1.6 (0.5)	No statistical difference
Patel et al [29]	2.23	2.33	No statistical difference
Vaira et al [31]	4	ChatGPT: 4; Bard: 3	No statistical difference
5-point Likert scale			
Currie et al [24]	1.9	2.8	47
Gondode et al [10]	4.5 (0.2)	4.5 (0.2)	No statistical difference
Shiraishi et al [30]	5	3.9 (0.5)	No statistical difference

a FKGL: Flesch‐Kincaid Grade Level.

b FRES: Flesch Reading Ease Score.

c GFI: Gunning-Fog Index.

d SMOG: Simple Measure of Gobbledygook.

e CLI: Coleman-Liau Index.

### AI-Assisted Consent Acquisition

Five (15.2%) papers explored AI-assisted consent acquisition, employing a wide range of methodologies and evaluating various outcomes.

Teasdale et al evaluated participants’ comprehension, satisfaction, and trust in GPT-4 as a substitute for human-led informed consent. In a 15- to 20-minute simulated consultation, participants, including laypeople and medical staff, engaged with the platform to ask questions and clarify doubts about a surgical procedure [22]. Feedback was generally positive but cautious; 71% felt sufficiently informed and capable of making an informed decision, 86% considered that the provided information was clear, and 57% felt respected and would recommend the process. While AI was praised for standardizing information and reducing errors, concerns about its lack of human empathy and data privacy were highlighted.

Building on this, Aydin et al [18] conducted an RCT comparing patient satisfaction and understanding of coronary angiography between those receiving traditional physician-led consent and those using GPT-3.0 to ask questions about the procedure. While patient satisfaction levels were similar between the 2 groups (*P*=.58), the AI-assisted group demonstrated a significantly better comprehension of coronary angiography risks (*P*<.001).

Similarly, Chung et al [19] assessed the effectiveness of GPT-4.0 in prevasectomy counseling through another RCT, comparing patients who received standard in-person consultations to those who engaged with ChatGPT before their appointment. While both groups reported high satisfaction, the AI-assisted group demonstrated significantly improved provider-perceived patient understanding of the procedure (8.8±1.0 vs 6.7±2.8; *P*=.047) and required shorter consultation times (7.7±2.3 min vs 10.6±3.4 min; *P*=.05).

Further supporting AI’s role in informed consent, Gan et al [20] conducted an RCT evaluating ChatGPT-assisted consent in total knee arthroplasty. Compared to traditional consent, the AI-assisted group experienced significantly lower anxiety levels both after consent (Hospital Anxiety and Depression Scale–Anxiety subscale: 10.48±3.84 vs 12.75±4.12; *P*=.04) and on the fifth postoperative day (8.33±3.20 vs 10.71±3.83; *P*=.01). They also reported greater satisfaction with preoperative education (4.22±0.51 vs 3.43±0.84; *P*<.001).

## Discussion

### Principal Findings

This systematic review examined the role of LLMs in enhancing the informed consent process and transforming patient education. With extensive training on vast datasets and optimization for human-like language generation, these models have demonstrated significant potential to improve the clarity, accessibility, and comprehensiveness of patient information across various medical specialties [6,48,49]. Our review identified 33 studies exploring this application, categorized into 3 main areas: LLMs responding to patient inquiries, generating consent documents, and directly enhancing the informed consent process, either as a supplement to physician interactions or as a stand-alone tool.

Among the identified studies, more than half (n=21, 63.6%) were cross-sectional or comparative cross-sectional, indicating that AI research in medical consent remains largely in an observational stage. Nevertheless, the emergence of RCTs suggests a growing integration of AI into clinical practice, with an increasing number of high-quality, intervention-based studies incorporating this technology into the literature. Additionally, research on this topic is predominantly conducted in North America and Europe, while contributions from Asian countries, including China, Japan, and India, remain comparatively limited. Notably, the majority of studies (n=22, 64.7%) report no external funding, highlighting the feasibility of assessing AI applications without reliance on financial support from external grants.

The majority of included studies focused on patient education, where AI tools such as ChatGPT were tasked with answering patient inquiries to enhance understanding. Overall, the findings showed that AI models can provide accurate information that aligns with approved materials currently in use [17,35,37]. However, concerns about readability were raised in many of these studies. The National Institutes of Health recommends that health-related materials for the general public should aim for an FRES of 60 or higher [50]. While AI models generally exceed this score, so do many commonly used informational pamphlets in health care settings [34,50-52].

Nevertheless, LLMs possess the capability to adjust their reading level to optimize readability, suggesting that this issue can easily be addressed. As evidenced by Shah et al [1], when asked to do so, ChatGPT adjusted its response to a grade 7.5 reading level, slightly below the required grade 8, without compromising accuracy [42]. This was further supported by studies on AI-driven conversational platforms used in the creation of consent documents, where most found that AI-generated documents had better readability than current consent forms, while still maintaining a high level of informational quality [23,25,31].

In the context of AI-assisted consent acquisition, AI was employed to interact with patients in real time, providing explanations of procedures. These studies, mostly RCTs, found promising results, indicating that patients who interacted with AI-driven conversation platforms demonstrated a higher level of understanding compared to those receiving traditional informed consent from specialists, while also reporting higher satisfaction levels with their experience [18,19,21]. Furthermore, reductions in anxiety levels were described by Gan et al [20] in patients using this approach, both after consent and on the fifth postoperative day.

### Reconfiguration of Communication Dynamics

Beyond its impact on clinical outcomes, AI-assisted informed consent has the potential to reshape the communicative structure of the consent encounter itself. Traditional models of shared decision-making conceptualize informed consent as a dyadic interaction in which information exchange and deliberation occur between clinicians and patients [53]. Similarly, the 3-talk model describes the process as progressing through team talk, option talk, and decision talk [54].

The introduction of an LLM effectively adds a third communicative actor, transforming the interaction into a patient-clinician-AI triad and redistributing communicative responsibilities. In this context, AI may assume much of the information-delivery function associated with “option talk,” while clinicians are able to focus more directly on values clarification, deliberation, emotional support, and decision-making. Interpreted through the patient-centered communication framework, which encompasses information exchange, relationship building, uncertainty management, and emotional responsiveness [55], the studies included in our review suggest that AI may enhance the informational dimension of communication, as reflected by improvements in patient comprehension, while potentially allowing clinicians to devote greater attention to relational and decisional aspects of care.

Despite the potential benefits, several areas need refinement before AI models can be fully integrated into clinical practice, with data privacy being a key concern. AI technologies rely on the processing of large volumes of personal data, and patients often lack a clear understanding of how their information is being used [22,56]. However, this issue is not exclusive to AI and is also a challenge with many electronic health record systems. One possible solution is to apply the principles of the General Data Protection Regulation, which governs current software systems, to AI technologies [22]. This would ensure that the same strict privacy standards are upheld when obtaining patient consent for the use of AI models.

Beyond regulatory compliance, AI-mediated consent raises information-ethics questions specific to health communication, in which accessibility, accuracy, and accountability carry ethical weight alongside confidentiality [57]. A central concern is equity of access. Because AI consent tools presuppose digital access and competence, they risk reproducing both first-level and second-level digital divides [58] and the uneven distribution of eHealth literacy [59], disadvantaging patients with low functional health literacy, older adults, low-income groups, and those with limited English proficiency, precisely the populations for whom the comprehension of consent is already most precarious. Informatics interventions of this kind can inadvertently widen the disparities they are intended to reduce when their benefits accrue disproportionately to more advantaged users.

Furthermore, while the information provided by LLMs is generally accurate, it is important to note that some references they cite can be incorrect, outdated, or even fabricated [17,39]. The precise and detailed functioning of LLMs remains unclear, with the only certainty being that all input data are integrated and processed to generate human-like responses [56,60]. This phenomenon is often referred to as the “black box of AI,” a significant challenge in computer science. The question of whether this black box can ever be fully opened and its processes fully understood remains unresolved [60]. No solutions to this issue have yet been established, but numerous studies emphasize the need for appropriate regulatory frameworks, robust quality control standards, and careful validation of the technology [56-60].

In the context of health communication, the “black box” problem primarily reflects a lack of transparency in how AI-generated information is produced. Merely acknowledging this limitation is insufficient; transparency must be supported by practical safeguards. Transparency has been defined as the combination of intelligibility and accountability and is a fundamental requirement of trustworthy health AI [56,61]. In practice, this can be operationalized through specific disclosure measures, including clear labeling of AI-generated content, informing patients when AI has been used to prepare consent materials, documenting the model’s identity and version, and providing source citations that have been verified by a clinician to mitigate the risk of fabricated references.

Additional safeguards include incorporating a plain-language explanation of the AI system’s role and limitations within consent documents, requiring clinician review and sign-off before use, documenting this oversight in the medical record, and ensuring that patients retain the option to receive information supervised by or directly from a health care professional. Framed in this way, transparency becomes a measurable and enforceable component of the informed consent process rather than an abstract ethical principle.

### The Need for Human Oversight

Despite the promising performance of AI in the informed consent process, human clinical oversight remains an indispensable component. Informed consent is not merely an information exchange; it is a legal and ethical process that requires clinical judgment, contextual sensitivity, recognition of patient vulnerability, and empathic communication—capacities that current AI systems cannot reliably replicate. Even when LLMs demonstrate high aggregate accuracy, the risk of generating inaccurate, fabricated, outdated, or contextually inappropriate content persists and has been directly documented in the literature included in this review [41,42]. These findings highlight a critical discrepancy between quantitative performance metrics and the real-world reliability standards required for safe clinical communication. Furthermore, a significant proportion of patients may experience AI-assisted consent as impersonal or inadequate, raising concerns about patient dignity and the therapeutic relationship, both of which are recognized as integral to ethically valid consent [25].

Accordingly, the current evidence supports a model in which AI functions as a supplementary tool to enhance, rather than replace, human-led informed consent. AI may appropriately serve to prepare patients prior to a clinical encounter, improve the readability and comprehensiveness of written consent documents, and address common procedural questions in a standardized and accessible format. However, the final consent conversation, including the opportunity for patients to ask individualized questions, express concerns, and confirm genuine understanding, must remain under the supervision of a qualified clinician who bears legal and ethical responsibility for the consent process.

Furthermore, informed consent is not merely the transfer of information but a fundamentally relational process in which trust plays a central role. Clinical empathy is a key component of this relationship and has been associated with improved patient outcomes [62,63]. Although current LLMs can generate responses that patients perceive as empathic and, in some contexts, have been rated as more empathetic than physician responses [64], this apparent empathy does not reflect genuine understanding or accountability. Consequently, reliance on AI for consent discussions introduces unique challenges. Excessive delegation of consent communication to AI may weaken the therapeutic relationship, particularly if patients later discover that expressions of empathy were algorithmically generated. These concerns support the view that AI should serve as an adjunct rather than a substitute for clinician-led informed consent. While AI may enhance the informational aspects of the consent process, the empathic, relational, and values-based elements, as well as ultimate responsibility for the discussion, should remain with the clinician. A particularly promising approach is a “warm handoff” model, in which AI-generated materials are reviewed, contextualized, and personalized through direct clinician-patient dialogue.

### Implications for Health Communication and Policy

Beyond the individual clinician-patient encounter, the findings of this review have important implications for the delivery of health communication at the system level. If appropriately validated and governed, AI-assisted consent tools could serve as a standardized communication infrastructure, providing consistent, plain-language procedural information across clinicians, departments, and institutions. Such tools have the potential to reduce the well-documented variability in consent quality while expanding communication capacity in high-volume, resource-constrained, and geographically remote settings where clinician time is limited. The studies included in this review suggest that AI-assisted consent may improve patient comprehension and satisfaction while reducing consultation time, indicating potential gains in both efficiency and patient-centered care. These benefits align with the equity-focused Quintuple Aim of health care: improving patient outcomes, patient and clinician experience, health equity, and system efficiency while reducing costs [65]. At the population level, AI-generated materials optimized for readability and held to established reading-level standards could support functional health literacy, a recognized public health objective [66,67]. In this context, informed consent may evolve from a one-time event into an ongoing, supported communication process.

Realizing these benefits, however, requires robust governance. At the institutional level, health care organizations should establish clear policies governing the use of AI in informed consent. These policies should require clinician verification and documented human oversight of AI-generated content, adoption of readability standards (targeting a sixth-grade reading level), routine auditing of accuracy and readability, and safeguards to promote equity, including multilingual materials, plain-language defaults, and nondigital alternatives. Institutions should also support clinician training in AI-assisted communication and invest in initiatives that strengthen patient digital and eHealth literacy. Ongoing postdeployment monitoring, incident reporting, and version control should form part of standard governance processes.

At the regulatory and public health levels, oversight frameworks should be proportionate to the risks associated with AI-generated consent materials and aligned with international guidance on the ethics and governance of health AI [59]. Key priorities include transparency requirements regarding the use of AI and the development of standardized reporting guidelines and outcome measures for future research. Such standardization is particularly important, given the methodological heterogeneity that prevented quantitative synthesis in the present review. Policymakers should also incorporate AI-related communication competencies into national health-literacy strategies and implement population-level monitoring to ensure that these technologies reduce, rather than exacerbate, existing health disparities. Integrating AI-assisted consent within established health-literacy and shared decision-making frameworks, rather than treating it as a stand-alone technological intervention, is likely to maximize its potential to improve the quality, accessibility, and equity of informed consent.

### Study Limitations

An important limitation of this review is that a meta-analysis could not be conducted because of substantial methodological heterogeneity across the included studies. This was primarily due to the lack of standardized definitions and outcome measures across studies. Effectiveness was evaluated in diverse ways, encompassing outcomes such as accuracy, readability, completeness, relevance, comprehension, satisfaction, and anxiety, which were assessed using a wide range of nonstandardized instruments and scoring systems. Furthermore, even when similar outcomes were evaluated, studies frequently employed different metrics, scales, or evaluation methods, limiting comparability across studies and preventing meaningful quantitative synthesis of the findings.

Furthermore, although our search strategy was not restricted by medical specialty, the included studies were predominantly surgical in focus. The absence of pediatric, mental health, and other nonsurgical specialties likely reflects the early stage of AI adoption in those fields, the heightened ethical complexity surrounding consent in vulnerable populations, and the more relational nature of consent processes in nonprocedural specialties. Future research should prioritize these underrepresented areas.

### Conclusion

This review highlights the potential of LLMs to improve the informed consent process and patient education, particularly by enhancing accuracy and comprehensiveness. However, challenges related to the reliability of references and ethical concerns must be addressed before their full adoption. Continued advancements in AI models, along with rigorous research into the ethical integration of AI in clinical settings, will be essential for ensuring the safe and effective incorporation of this technology into health care.

## Supplementary material

10.2196/93501 Checklist 1 PRISMA checklist.

## Abbreviations

FKGL: Flesch-Kincaid Grade Level
FRES: Flesch Reading Ease Score
LLM: large language model
PRISMA: Preferred Reporting Items for Systematic Reviews and Meta-Analyses
PROSPERO: International Prospective Register for Systematic Reviews
RCT: randomized controlled trial
RoB2: Cochrane Risk of Bias Tool 2
